# Development of the Hypopharyngeal Glands of Worker Bees (*Apis mellifera* L.) When Fed Different Protein Sources During the Spring Period

**DOI:** 10.3390/insects17010021

**Published:** 2025-12-23

**Authors:** Svilen B. Lazarov, Ivaylo G. Georgiev, Atanas Z. Atanasov, Ivaylo S. Hristakov

**Affiliations:** 1 Department Livestock-Non-Ruminant Animals and Special Industries, Trakia University, Students Campus, 6000 Stara Zagora, Bulgaria; svilen.b.lazarov@trakia-uni.bg (S.B.L.); ivaylo.georgiev@trakia-uni.bg (I.G.G.); 2 Department of Agricultural Machinery, Agrarian and Industrial Faculty, University of Ruse, Angel Kanchev, 7017 Ruse, Bulgaria; ihristakov@uni-ruse.bg

**Keywords:** honey bee, HPGs, protein sources, supplementary feeding

## Abstract

The supplementary feeding of bee colonies under conditions of limited natural food sources is essential for maintaining their health and productivity. Pollen is a major source of protein for bees, collected during plant flowering. Its absence negatively affects the secretory activity of the hypopharyngeal glands (HPGs) in young nurse bees. This study aimed to assess the effect of different protein sources provided during the spring period on the development of HPGs in worker bees. An experiment was conducted with 28 bee colonies divided into seven groups of four colonies each—one control and six experimental. The colonies were fed soy isolate (*Glycine max*), brewer’s yeast (*Saccharomyces cerevisiae*), spirulina (*Arthrospira platensis*), pea protein (*Pisum sativum*), and bee pollen (a mixture of pollens from various plant species). The results confirmed the critical role of pollen, as 40% of bees in the pollen-fed group exhibited HPGs at grade 4. The group supplemented with spirulina showed similar results (35.8%), indicating its potential as an alternative protein source. These findings highlight the importance of proper protein supplementation and suggest that spirulina could serve as a promising substitute for pollen in early spring feeding to support colony development.

## Simple Summary

Honey bees need good protein nutrition in early spring to develop strong nurse bees and raise healthy broods. When natural pollen is scarce, beekeepers use protein supplements, but their effectiveness varies. In this study, we compared six protein sources to see how they affect the development of the hypopharyngeal glands in worker bees. Bee pollen produced the best results, confirming its role as the most complete natural protein. Spirulina showed almost the same effectiveness and appears to be a strong alternative when pollen is limited. Soy isolate and pea protein had moderate positive effects, while brewer’s yeast was the least effective. These results can help beekeepers choose the most suitable protein supplements to support colony development during early spring.

## 1. Introduction

Honeybees obtain their nourishment from the flowers of plants. The pollen they collect and process into bee pollen serves as a natural source of proteins, vitamins, minerals, lipids, and enzymes, whereas nectar provides carbohydrates essential for energy metabolism [1,2].

Globally, climate change has led to periods of pollen shortages in natural ecosystems, which in turn result in deficiencies of proteins and amino acids in the diet of bee colonies. According to several authors, the impact of pollen deficiency on honey bee nutrition varies depending on the subspecies involved [3]. In their study, the authors examined how pollen feeding affects two subspecies endemic to Algeria: *Apis mellifera intermissa* and *Apis mellifera sahariensis*. Two groups were formed within each subspecies: one group of newly emerged bees housed in cells received pollen as food, whereas the other did not.

The authors reported that, in the *Apis mellifera sahariensis* group deprived of pollen, the bees lived on average 5.5 days longer compared with individuals of *Apis mellifera intermissa* that received pollen. Furthermore, the hypopharyngeal glands of worker bees from the sahariensis group were more developed and less affected by pollen deprivation than those of intermissa under identical conditions.

Liang et al. [4] reported that the type of pollen influences longevity, live weight, proteolytic enzyme activity in the midgut, and hypopharyngeal gland development in worker bees. In their study, the authors evaluated the effects of four types of pollen—oilseed rape, camellia, lotus, and buckwheat pollen. They found that diets containing oilseed rape or camellia pollen had the strongest positive effect on hypopharyngeal gland development and proteolytic enzyme activity in the midgut.

According to Corby-Harris et al. [5], pollen consumption in bees is positively correlated with the development of their hypopharyngeal glands. Up to three days of age, the development of the hypopharyngeal glands is similar among individuals, and nutrient availability exerts a greater influence after the third day. The authors conclude that hypopharyngeal gland development during the first three days of life in worker bees is a predetermined physiological process.

In contemporary apicultural practice, supplementary feeding is increasingly implemented to compensate for nutrient deficiencies or to address the insufficient quality of available natural food sources. Numerous studies have demonstrated that protein-enriched diets positively influence various parameters related to colony growth and development, including brood area, population strength, and overall vitality.

In beekeeping practice, a variety of protein sources have been tested as nutritional supplements to enhance colony nutrition, such as soybean meal [6], brewer’s yeast [7], skimmed milk [8], wheat gluten [9], chickpea flour [10], spirulina algae (*Arthrospira platensis*) and *Chlorella* sp. [11,12,13,14].

Mohamed et al. [15] conducted an experiment in which honey bees were fed with extracts of chamomile (*Matricaria chamomilla*), caraway (*Carum carvi*), sesame (*Sesamum indicum*), anise (*Pimpinella anisum*), bay laurel leaves (*Laurus nobilis*), and ginger (*Zingiber officinale*). The authors found that feeding bees with anise, bay laurel, and ginger had a particularly positive effect on the development of their hypopharyngeal glands.

Several authors have investigated the effects of stimulative and nutritive products of both plant and other origins, determining appropriate doses and methods of application in bee colonies [16,17,18,19]. According to [2], complete and balanced nutrition is a key factor in maintaining healthy and resilient bee colonies. Similarly, DeGrandi-Hoffman et al. [20] established a correlation between food composition, protein levels, and the immune response of bees, emphasizing that colony losses caused by protein deficiency can be mitigated through adequate supplementary feeding.

Honeybees have evolved complex mechanisms of adaptation to changing environmental conditions. Within a colony, there are two sexes—males (drones) and females (queens and workers). The female caste is divided into queens and workers, which differ behaviorally, morphologically, and physiologically. Worker bees exhibit age polyethism, performing different tasks throughout their lifespan, such as cleaning comb cells, nursing the brood, storing food, foraging, and guarding the hive [21]. These behavioral transitions are accompanied by physiological changes, including structural modifications of glands, variations in secretory products, and alterations in neuroanatomy and neurochemistry [22].

One of the most prominent physiological changes during the transition from nurse to forager is the alteration in the secretory activity of the hypopharyngeal glands (HPGs). In worker bees (*Apis mellifera* L.), the HPGs are paired structures located in the anterior part of the head between the compound eyes. Each gland consists of multiple secretory units (acini) connected by a central duct [23,24]. The primary products of HPG secretion are royal jelly and enzymes such as αβ-glucosidase. The glands reach peak development at approximately six days of age; thereafter, the acinar volume and the number of secretory vesicles gradually decrease, and degenerative changes occur in forager bees [25,26]. According to Maurizio [23], the condition of the HPGs can serve as a physiological indicator of the nutritional status and social role of worker bees. Furthermore, their activity reflects the overall state and needs of the colony—when nurse bees are lacking, older workers may reactivate their glands and resume royal jelly secretion [27].

Previous studies have demonstrated correlations between the degree of HPG development and the application of various carbohydrate and protein sources in bee feeding [28,29,30,31,32,33,34,35]. However, despite the increasing amount of research on supplementary feeding, information regarding the comparative effects of different protein sources on the histological and functional development of the hypopharyngeal glands in worker bees remains limited and often inconsistent, particularly under spring conditions. Moreover, data concerning the effectiveness of alternative protein supplements derived from plants and microalgae remain fragmented. Addressing this knowledge gap is essential for identifying sustainable, nutritionally efficient, and economically viable substitutes for natural pollen in apicultural practice.

The aim of the present study was to evaluate the influence of feeding bee colonies with different protein sources during the spring period on the development of the hypopharyngeal glands (HPGs) in worker bees.

## 2. Materials and Methods

### 2.1. Study Site

The study was conducted at an apiary located in the village of Korkina, Kyustendil District, Republic of Bulgaria (42°18′33.48″ N, 22°57′33.84″ E), at an altitude of 750 m, classified as a semi-mountainous area. The site is characterized by numerous early-flowering plant species, including hazel (*Corylus avellana*), cornel (*Cornus mas*), blackthorn (*Prunus spinosa*), and cherry plum (*Prunus cerasifera*), which provide pollen from late winter to early spring (late February–early March). The area also contains extensive orchards, mainly cherry (*Prunus avium* L., cultivated varieties), European plum (*Prunus domestica*), and apple (*Malus domestica*), which typically reach peak flowering in the first ten days of April, supplying substantial amounts of pollen and nectar until early May.

During the experimental period, atypical climatic conditions occurred, characterized by a prolonged period of low temperatures both day and night. Particularly critical were nighttime temperatures ranging from −8 to −6 °C, which persisted for nearly a week and resulted in the destruction of 90–100% of flowers and flower buds of all blooming plants, including orchard species in the “bud” or “early bloom” phenophases. Consequently, the bees were deprived of natural pollen and nectar for an extended period. The damage was so extensive that on 11 April 2025, a state of emergency was declared for the entire Kyustendil District, according to the official order of the regional governor (Order OA-RD-14-66/11.04.2025) [36].

This event represents a real and acute, sharply occurring, and verifiable pollen shortage during the spring season in which the field experiment was conducted. The resulting conditions can be considered a natural, unplanned “stress test” for the bee colonies, in which the role of supplemental protein feeding became particularly important.

### 2.2. Experimental Design and Grouping of Bee Colonies

The experiment was carried out with 28 honeybee colonies (*Apis mellifera* L.) housed in 10-frame Dadan-Blatt hives. The queen bees were selected from the same maternal line (sister queens). Prior to the beginning of the feeding trial, all colonies were equalized in terms of strength, amount of sealed brood, and stores of honey and bee pollen.

The strength of the bee colonies was assessed using the method described by [37], by counting the frames fully occupied by bees. In a Dadant-Blatt hive, one frame corresponds to approximately 0.250 kg of bees.

The area of stored pollen (bee bread) was measured using a calibrated frame according to [37]. The quantity of capped honey was determined with a calibrated frame following [38], and the area of capped brood was measured using a calibrated frame according to the method described by [39].

Seven groups of four colonies each were formed—one control group and six experimental groups.

The following protein sources were used for the experimental groups: soy isolate (*Glycine max*) obtained from Vivena Ltd. (Stroyevo, Bulgaria), brewer’s yeast (*Saccharomyces cerevisiae*) sourced from Buldaikhim Ltd. (Rakititsa, Bulgaria), spirulina (*Arthrospira platensis*) provided by Burel Organics (Sofia, Bulgaria), pea protein (*Pisum sativum*) acquired from Bionia Ltd. (Pernik, Bulgaria), and bee polyfloral pollen (a mixture of pollens from various plant species) collected in spring 2024. The pollen was dried and stored in a hermetically sealed container until the start of the experiment. The target crude protein content of the diets for the experimental groups was 20%, while the diet provided to the control group contained no protein. The powdered sugar used was obtained by finely grinding white crystalline cane sugar. The inverted sugar syrup used in the preparation of the diets was a commercial product, APIINVERT^®^ (Südzucker AG, Mannheim, Germany).

The content of the main nutrients in the protein sources used was determined through laboratory analyses conducted at the Research Laboratory of the Faculty of Agriculture, Trakia University, Stara Zagora, Bulgaria, using the following methods:

➢ Moisture (%)—Gravimetric analysis, BSS-ISO 6496:2000
➢ Crude ash (%)—Gravimetric analysis, BSS-ISO 5984:2022
➢ Crude protein (%)—Kjeldahl method, BSS-EN ISO 5983-1:2006
➢ Crude fat (%)—Soxhlet extraction, BSS-ISO 6492:2007
➢ Crude fiber (%)—Gravimetric analysis according to Henneberg and Stohmann, VVLM 3/2024 (Internally validated laboratory method)

The results of these analyses are presented in Table 1.

The crude protein content of the food for the experimental groups was 20%, whereas the control diet contained no protein. Table 2 presents the ingredients and their respective quantities used to prepare 1 kg of each diet. The ingredients were thoroughly mixed until a dough-like consistency was achieved. The prepared diets were portioned into rations of 0.200 kg, packaged in polyethylene bags, and stored at 5 °C. Prior to placement in the hives, the food rations were tempered at room temperature for 24 h.

Each bee colony received six rations of 0.200 kg, administered once every seven days during the period from 10 March to 21 April 2025. The food rations were placed on the upper slats of the frames within the hives.

### 2.3. Determination of Hypopharyngeal Gland (HPG) Developmental Stages

To assess the stages of hypopharyngeal gland (HPG) development, 30 randomly selected young, non-flying worker bees from the nurse bee caste were collected from each colony after the completion of feeding, resulting in a total of 840 bees. To obtain the sample, bees from a frame with open brood were gently brushed off using a bee brush onto a 50 × 50 cm sheet of paper placed on the ground in front of the hive. After 1–2 min, the sheet was shaken, leaving only the non-flying young nurse bees, while older foraging bees flew away and returned to the hive. In this way, a sample of worker bees was obtained that provides general information on the average development of the hypopharyngeal glands (HPGs) of the nurse bee caste under field conditions.

For HPG preparation, bees were anesthetized using diethyl ether. A horizontal incision was made through the head with a razor blade. The glands were carefully extracted with fine tweezers and placed in a drop of distilled water on a glass slide.

The hypopharyngeal glands (HPGs) were examined using a trinocular light microscope VEVOR XSP-36TV (VEVOR, Shanghai, China), configured with a 4× objective and a WF25× eyepiece (total magnification 100×). A color filter was placed in the illumination system. The assessment of HPG development was performed directly during microscopic observation. Demonstration images of approximately 5% of the examined bees were captured using a digital microscope camera MY SCOPE, model MY-01 (MY SCOPE, Shenzhen, China), mounted on the trinocular port via a 0.5× optical reducer, at a resolution of 1920 × 1080 px (see Supplementary Figure S1).

HPG development was evaluated using a four-grade scale according to Maurizio [23].

Grade 1—Main and side ducts clearly visible; acini underdeveloped, irregular, often nodular, and transparent.

Grade 2—Ducts visible; acini irregularly rounded, with clear intercellular spaces, still transparent.

Grade 3—Main duct visible; side ducts obscured by partially developed acini, which are less transparent.

Grade 4—All ducts completely covered; acini densely arranged, maximally developed, turbid-white or yellowish.

### 2.4. Statistical Analysis

The distribution of HPG developmental levels across the experimental groups was analyzed using the Chi-square (χ^2^) test. This test was applied to determine whether a statistically significant association exists between the type of feeding and the frequencies of HPG development levels.

To assess significant differences in the distribution of HPG developmental stages among groups, the Kruskal–Wallis H test was performed. Pairwise comparisons between groups were conducted using the Mann–Whitney U test to identify which groups differed significantly.

The effect size of feeding on HPG development was estimated by calculating the r-coefficient according to the following formula:

r = z/(√N)(1)

where:

z—standardized normal value derived from the Mann-Whitney U test statistic. indicates the extent of differences between the compared groups:

N—total sample size.

A *p*-value of <0.05 was considered statistically significant. Statistical analyses were performed using the SPSS Statistics Version 19.0; IBM Corp.: Armonk, NY, USA, 2010 [40].

## 3. Results and Discussion

The content of the main nutrients in the prepared diets was determined by laboratory analysis conducted at the Research Laboratory of the Faculty of Agriculture, Trakia University, Stara Zagora, following the methods described in the “Materials and Methods” section. The results of the analysis are presented in Table 3.

Table 4 presents the mean values and standard deviations per group for capped honey, bee bread, sealed brood, and colony strength prior to the start of the experimental feeding. Kruskal–Wallis tests revealed no statistically significant differences (*p* > 0.05), confirming that the colonies were successfully equalized before the experiment.

Table 5 presents the distribution of HPG developmental levels, as assessed by the χ^2^ (Chi-square) test. Cramér’s V coefficient (V = 0.120; *p* < 0.001) indicates a weak to moderate, statistically significant association between HPG developmental levels and the experimental groups.

In all experimental groups, the proportion of bees exhibiting grade 4 HPG development was higher than in the control group. The highest percentage (40%) was observed in the group fed with bee pollen, followed by the group supplemented with spirulina (35.8%). In the control group, only 16.7% of the bees reached grade 4 development, which is more than two times lower than in the bee pollen and spirulina groups. These results indicate a positive effect of supplementing the bee diet with the respective protein sources. Similar findings have been reported in recent studies, confirming the beneficial impact of protein-rich diets on hypopharyngeal gland development in honey bees [14].

Analysis of the results presented in Table 5 revealed that, for the third stage of hypopharyngeal gland (HPG) development in worker bees, the highest proportion of bees (46.7%) was observed in the control group. This may be attributed to the high carbohydrate content in their diet and the availability of natural pollen. In the group of colonies supplemented with spirulina, 45.8% of bees reached stage 3, which was higher than in the other experimental groups. Supplementation with spirulina appears to support the development of bee colonies.

Previous studies have reported that diets containing spirulina increase thorax weight, elevate protein content in the head, and enhance beneficial gut microbiota compared with other protein sources, despite lower overall consumption of spirulina-containing diets [13]. These authors also demonstrate that spirulina has significant potential as a feed supplement or pollen substitute, exerting multiple positive effects on the physiology of nursing worker bees. Furthermore, supplementation with spirulina significantly increases lipid content in worker bees and elevates levels of the lipoprotein vitellogenin [13].

Regarding stage 2 HPG development, the highest proportion of bees (30.0%) was observed in both the control group and the brewer’s yeast group. In stage 1, the greatest proportion of bees (8.3%) was recorded in the group fed with pea protein.

The Kruskal–Wallis test (χ^2^ = 23.869, df = 6, *p* = 0.001) revealed statistically significant differences among at least two of the groups, indicating the need for subsequent pairwise comparisons. All possible pairwise comparisons were performed using the Mann–Whitney U test (Table 6).

The statistically significant differences were primarily observed between the experimental groups and the control group, although some differences were also detected among the experimental groups themselves. Supplementation with spirulina or bee pollen resulted in a significant increase in HPG development compared to the group fed with brewer’s yeast, indicating a comparatively weaker effect of brewer’s yeast in this experiment. Nevertheless, brewer’s yeast still produced a higher average HPG development than the control group, consistent with the findings of [42]. Previous studies have also demonstrated the positive effects of spirulina supplementation on colony performance, including increased colony strength and the quantity of sealed brood [43], supporting the beneficial role of this protein source in apicultural practice.

Spirulina, brewer’s yeast, and bee pollen were also included in the diet of group 6. The comparatively weaker effect of brewer’s yeast on HPG development in this context likely influenced the overall outcome, as evidenced by the statistically significant difference between group 5 (bee pollen) and group 6 (mixture). A similar pattern is observed in the absence of statistically significant differences relative to the control group in the two experimental groups containing brewer’s yeast (groups 2 and 6).

The highest effect size of feeding (r = 0.252) was observed for bee pollen, confirming its role as an essential protein source for honey bees. The effect of spirulina as a single protein source was also notable, with the second largest effect size (r = 0.242), nearly equivalent to that of bee pollen. These findings are consistent with the conclusions of [13], which emphasize the potential of spirulina as a complete or prebiotic dietary supplement. In addition, recent studies have shown that supplementation with the microalga Chlorella sorokiniana significantly enhances food consumption, longevity, hypopharyngeal gland development, muscle formation, and vitellogenin (Vg) gene expression compared to diets consisting solely of pollen or sugar [44], further supporting the beneficial role of microalgal supplements in honey bee nutrition.

Soy isolate and pea protein exhibited almost identical results, with both groups showing statistically significant differences compared to the control (*p* = 0.009 and *p* = 0.012, respectively). Using the four-point HPG rating scale, grades 3 and 4 were considered indicative of good development. In group 1 (soy isolate), 73.4% of bees achieved grades 3 and 4, while in group 4 (pea protein), the proportion was 73.3%.

Despite the anticipated synergistic effect of combining multiple protein sources, group 6, which was fed a mixture of spirulina, brewer’s yeast, and bee pollen, exhibited a statistically weaker effect compared to the group fed exclusively with bee pollen (group 5). This is supported by a statistically significant difference between the two groups (*p* = 0.036; r = 0.135), as well as by the lower proportion of bees at grade 4 in group 6 (27.5%) compared to group 5 (40%).

Furthermore, the absence of statistically significant differences between groups 2 (brewer’s yeast) and 6 relative to the control group underscores the comparatively weaker effect of brewer’s yeast, both as a standalone component and in combination with spirulina and bee pollen. These findings suggest that the inclusion of brewer’s yeast in colony feeding has a relatively limited impact on HPG development when combined with more effective protein sources such as bee pollen and spirulina. This interaction may restrict the potential synergistic benefits of mixed diets and warrants further investigation in future experimental studies.

These findings collectively highlight the critical role of protein supplementation in supporting the development of hypopharyngeal glands in worker bees, as well as the overall physiological condition of the colony. Notably, bee pollen and spirulina demonstrated the strongest positive effects, both in terms of HPG development and the observed effect sizes, emphasizing their potential as highly effective protein sources in apicultural practice.

While previous studies have investigated the impact of various protein-rich diets on bee physiology and colony performance [13,42,43,44], our study provides novel insights by directly comparing multiple protein sources—including soy isolate, pea protein, brewer’s yeast, spirulina, bee pollen, and their combination—under controlled spring feeding conditions. The comparative evaluation of both individual and mixed protein supplements offers a more detailed understanding of their relative effectiveness, which has been insufficiently addressed in earlier research.

From a practical perspective, these results have important implications for beekeepers seeking to optimize colony nutrition during periods of natural pollen scarcity. The data suggest that supplementation with high-quality protein sources such as bee pollen or spirulina can enhance HPG development, potentially improving brood rearing, colony strength, and resilience to environmental stressors. Conversely, the limited effectiveness of brewer’s yeast, both as a single supplement and in combination with other protein sources, highlights the need for careful selection of feed components to achieve the desired nutritional outcomes.

Overall, this study contributes new evidence supporting the development of nutritionally balanced, economically feasible, and sustainable feeding strategies in apiculture. By elucidating the differential effects of diverse protein sources on worker bee physiology, our findings can inform targeted interventions aimed at maintaining healthy and productive colonies, thereby enhancing both bee welfare and beekeeping productivity.

## 4. Limitations

Despite the valuable insights provided by the present study, several limitations should be acknowledged. First, the experiment was conducted during a single spring season with atypical climatic conditions, which led to a temporary scarcity of natural pollen. While this scenario represents a real-world stress test for bee colonies, it may limit the direct generalizability of the results to other seasons or regions with different environmental conditions.

Second, although colonies were provided with pre-prepared experimental diets under a controlled feeding regimen, free flight and access to natural pollen sources could not be entirely excluded. However, all experimental groups were exposed to identical environmental conditions, and the critical period of pollen scarcity coincided with the phase of intensive colony development, ensuring that observed differences reflect the effects of the protein supplements rather than external variability.

Third, the sample size, while adequate for evaluating hypopharyngeal gland (HPG) development, may limit the statistical power for detecting subtle differences in pairwise comparisons among experimental groups.

Fourth, the study focused primarily on HPG development as a proxy for nutritional and physiological status, without assessing long-term outcomes such as overall colony productivity, immunity, or overwintering success.

Finally, the effects of combining multiple protein sources in mixed diets, including potential synergistic or antagonistic interactions, require further investigation.

Despite these limitations, the study provides biologically meaningful and practically relevant insights into the effects of protein supplementation on honey bee colonies under field conditions, reflecting real beekeeping practices and the challenges posed by environmental stressors.

## 5. Conclusions

The results of the present study clearly demonstrate that the type of protein source used in spring feeding of honey bee colonies significantly influences the development of hypopharyngeal glands (HPGs) in worker bees. Feeding with bee pollen resulted in the highest degree of gland development and the largest effect size, confirming its role as a natural, complete, and highly effective source of protein. Spirulina supplementation also produced a notable positive effect, with outcomes nearly equivalent to those observed for bee pollen, highlighting its high biological value and potential as an alternative or partial replacement for natural pollen. Soy isolate and pea protein exhibited moderate yet significant effects on HPG development, indicating their suitability as accessible and economically viable protein supplements in apicultural practice. In contrast, brewer’s yeast showed a comparatively weaker effect, both as a standalone source and in combination with other protein supplements.

Based on the results obtained, the following conclusions can be drawn:

Bee pollen remains indispensable for protein nutrition during the spring period;

Spirulina can be recommended as a highly effective alternative protein source;

Soy isolate and pea protein represent practical and economically reasonable substitutes with proven positive effects;

Brewer’s yeast demonstrates limited effectiveness and should be used with caution in mixed protein formulations.

These findings provide new insights into the comparative efficacy of different protein sources under controlled spring feeding conditions, addressing gaps in previous research and offering evidence-based guidance for practical colony management. The promising results obtained with spirulina warrant further targeted investigations to determine optimal doses, combinations, and feeding periods. Future studies should also explore the long-term effects of different protein supplements on colony productivity, brood development, immunity, and overall health, as well as their potential interactions with environmental stressors. Such research will help to refine protein supplementation strategies, ensuring sustainable, efficient, and practical approaches to enhancing honey bee nutrition and colony performance.

## Figures and Tables

**Table 1:** Chemical composition of the protein sources used (% of dry matter).

Protein Source	Moisture (%)	Dry Matter (%)	Crude Protein (%)	Crude Fat (%)	Crude Fiber (%)	Ash (%)	NFE (%) *
Bee-collected pollen	4.61	95.39	26.12	7.70	4.06	2.72	54.79
Pea protein (*Pisum sativum*)	2.88	97.12	80.11	0.51	1.38	5.55	9.57
Spirulina (*Arthrospira platensis*)	6.07	93.93	63.73	0.47	1.83	9.54	18.36
Brewer’s yeast (*Saccharomyces cerevisiae*)	3.02	96.98	42.03	0.36	1.16	4.36	49.07
Soy protein isolate (*Glycine max*)	3.67	96.33	87.47	0.33	0.15	6.36	2.02

* NFE—Nitrogen-free extractives, calculated as the difference to 100%.

**Table 2:** Components for preparing 1 kg of food.

Ingredient	Powdered Sugar (kg)	Inverted Syrup (kg)	Soy Isolate (kg)	Brewer’s Yeast (kg)	Spirulina (kg)	Pea Protein (kg)	Bee Pollen (kg)
Groups							
Control	0.750	0.250					
Group 1	0.455	0.316	0.229				
Group 2	0.050	0.474		0.476			
Group 3	0.210	0.476			0.314		
Group 4	0.224	0.526				0.250	
Group 5		0.234					0.766
Group 6	0.086	0.395		0.159	0.105		0.255

**Table 3:** Composition of the administered diets.

Diet ID	Moisture (%)	Dry Matter (%)	Crude Protein (%)	Crude Fat (%)	Crude Fiber (%)	Ash (%)	NFE (%) *
D1	2.13	97.87	0.07	0.01	1.16	0.033	96.60
D2	10.50	89.50	19.93	0.38	1.18	1.13	66.88
D3	15.32	84.68	20.01	0.17	1.01	2.18	61.31
D4	13.59	86.41	20.02	0.13	0.95	3.01	62.30
D5	12.51	87.49	20.03	0.46	0.91	1.40	64.69
D6	11.58	88.42	19.84	2.46	1.52	2.08	62.52
D7	13.97	86.03	19.99	0.72	0.53	2.35	62.44

Note: D1—control; D2—soy protein isolate; D3—brewer’s yeast; D4—spirulina; D5—pea protein; D6—pollen; D7—mixed protein diet. * NFE—Nitrogen-free extractives, calculated as the difference to 100%.

**Table 4:** Results of colony equalization [41].

Groups		Capped Honey (kg)		Bee Bread (cm^2^)		Sealed Brood (Cells)		Colony Strength (kg)	
		Mean	SD	Mean	SD	Mean	SD	Mean	SD
Control		4.080	0.209	243.8	23.9	575	97	0.969	0.063
Group 1 Soy isolate		4.135	0.056	256.3	51.5	650	192	0.969	0.063
Group 2 Brewer’s yeast		4.189	0.203	243.8	37.5	575	171	0.969	0.063
Group 3 Spirulina		4.069	0.360	243.8	42.7	550	129	0.969	0.063
Group 4 Pea protein		4.025	0.094	243.8	12.5	500	141	0.938	0.125
Group 5 Bee pollen		4.091	0.219	262.5	43.3	625	171	0.969	0.157
Group 6 Brewer’s yeast, spirulina, bee pollen		4.124	0.295	268.8	51.5	525	189	1.000	0.102
Kruskal	Chi-Square	1.373		1.305		3.002		0.777	
–Wallis	df	6		6		6		6	
Test	Asymp. Sig.	0.968		0.971		0.809		0.993	

**Table 5:** Distribution of hypopharyngeal gland (HPG) developmental stages by group.

Groups		Degrees of HPG				Total
		1	2	3	4	
Control	Count	8	36	56	20	120
	% within Group	6.7%	30.0%	46.7%	16.7%	100.0%
Soy isolate	Count	9	23	47	41	120
	% within Group	7.5%	19.2%	39.2%	34.2%	100.0%
Brewer’s yeast	Count	7	36	48	29	120
	% within Group	5.8%	30.0%	40.0%	24.2%	100.0%
Spirulina	Count	8	14	55	43	120
	% within Group	6.7%	11.7%	45.8%	35.8%	100.0%
Pea protein	Count	10	22	48	40	120
	% within Group	8.3%	18.3%	40.0%	33.3%	100.0%
Bee pollen	Count	4	22	46	48	120
	% within Group	3.3%	18.3%	38.3%	40.0%	100.0%
Brewer’s yeast + Spirulina + Bee pollen	Count	5	31	51	33	120
	% within Group	4.2%	25.8%	42.5%	27.5%	100.0%
Total	Count	51	184	351	254	840
	% within Group	6.1%	21.9%	41.8%	30.2%	100.0%
Cramer’s V = 0.120; (*p*) = 0.006						

Note: Data are presented as the number and proportion of bees per group in the corresponding row.

**Table 6:** Mann-Whitney U test results for differences between groups.

		Group 1 Soy Isolate	Group 2 Brewer’s Yeast	Group 3 Spirulina	Group 4 Pea Protein	Group 5 Bee Pollen	Group 6 Mix
Control	Mann-Whitney U	5872.0	6790.0	5314.0	5932.0	5220.0	6272.0
	r	0.169	0.052	0.242	0.161	0.252	0.119
	Asimp.Sig.	0.009 **	0.417	0.000 **	0.012 *	0.000 **	0.066
Group 1 Soy isolate	Mann-Whitney U		6319.0	6746.0	7140.0	6602.0	6772.0
	r		0.112	0.058	0.008	0.076	0.054
	Asimp.Sig.		0.084	0.367	0.906	0.237	0.399
Group 2 Brewer’s yeast	Mann-Whitney U			5800.5	6379.0	5690.0	6709.0
	r			0.179	0.104	0.192	0.062
	Asimp.Sig.			0.006 **	0.107	0.003 **	0.333
Group 3 Spirulina	Mann-Whitney U				6686.0	7049.0	6260.0
	r				0.066	0.019	0.062
	Asimp.Sig.				0.307	0.763	0.333
Group 4 Pea protein	Mann-Whitney U					6542.0	6832.0
	r					0.084	0.047
	Asimp.Sig.					0.193	0.468
Group 5 Bee pollen	Mann-Whitney U						6142.0
	r						0.135
	Asimp.Sig.						0.036 *

Note: U-values, effect sizes (r) and *p*-values are presented for each pair of experimental groups. Statistically significant differences are indicated as *—*p* < 0.05 and **—*p* < 0.01.

## Data Availability

The data presented in this study are available on request from the corresponding author.

## Supplementary Materials

The following supporting information can be downloaded at: https://www.mdpi.com/article/10.3390/insects17010021/s1.

## Abbreviations

The following abbreviations are used in this manuscript:

HPGs	Hypopharyngeal glands
