# Supplementary figures and images for: Development of the Hypopharyngeal Glands of Worker Bees (Apis mellifera L.) When Fed Different Protein Sources During the Spring Period
Source: Insects. 2025 Dec 23;17(1):21. doi: 10.3390/insects17010021 (PMC12841754; doi:10.3390/insects17010021)

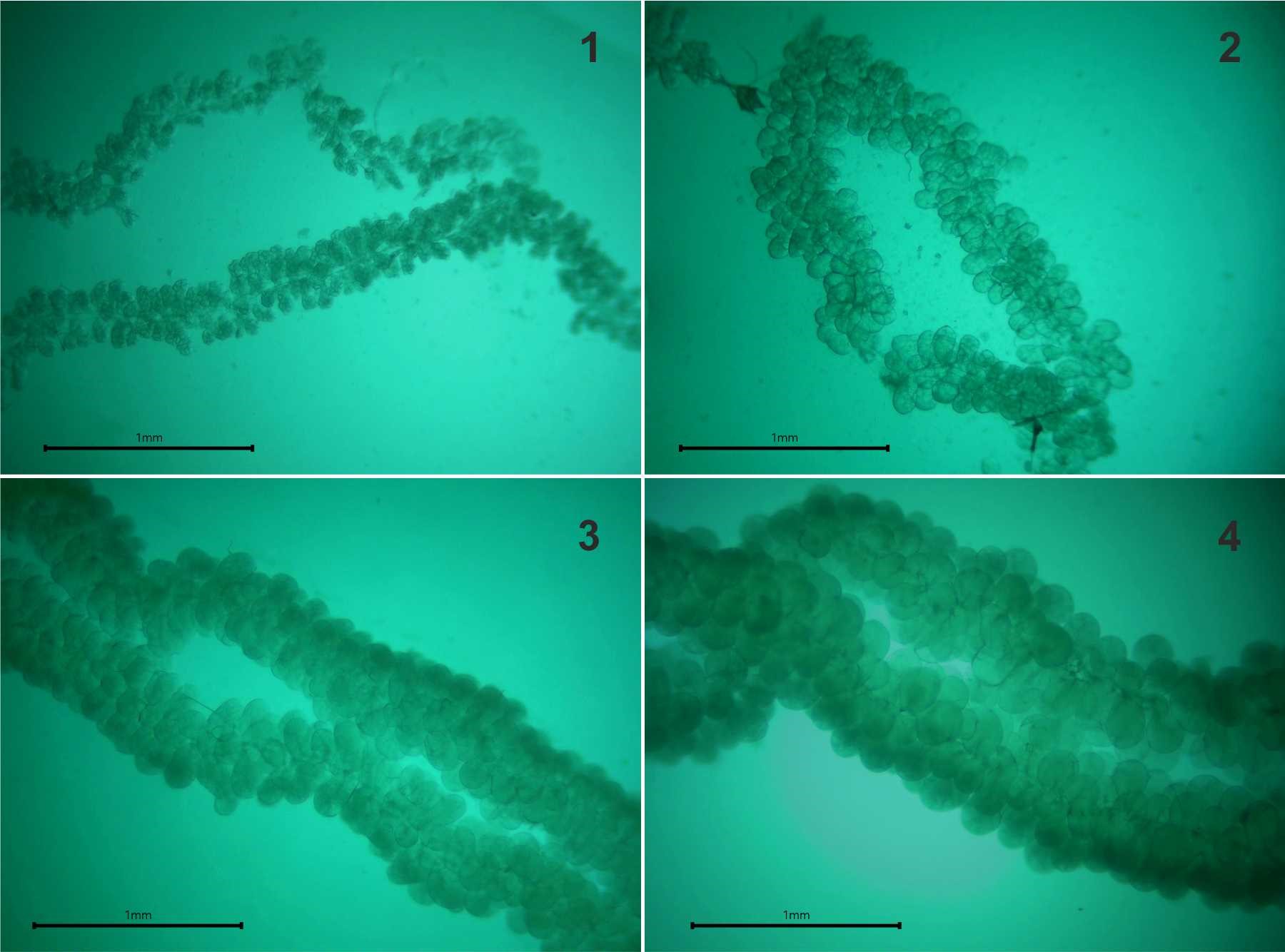

Supplement: Supplementary file 1 [file insects-17-00021-s001.zip › insects-4002126-supplementary/Stages of development of HPGs.jpg]
